# Artificial Intelligence and Machine Learning to Improve Evidence Synthesis Production Efficiency: An Observational Study of Resource Use and Time‐to‐Completion

**DOI:** 10.1002/cesm.70030

**Published:** 2025-05-19

**Authors:** Christopher James Rose, Jose Francisco Meneses‐Echavez, Ashley Elizabeth Muller, Rigmor C. Berg, Tiril C. Borge, Patricia Sofia Jacobsen Jardim, Chris Cooper

**Affiliations:** ^1^ Reviews and Health Technology Assessments, Division of Health Services Norwegian Institute of Public Health Oslo Norway; ^2^ Center for Epidemic Interventions Research, Division of Health Services Norwegian Institute of Public Health Oslo Norway; ^3^ Facultad de Cultura Física, Deporte y Recreación Universidad Santo Tomás Bogotá Colombia; ^4^ Tietoevry Norway AS Trondheim Norway; ^5^ Department of Community Medicine University of Tromsø Tromsø Norway; ^6^ Sensio AS Oslo Norway; ^7^ Bristol Medical School University of Bristol Bristol UK; ^8^ Department of Clinical, Educational and Health Psychology University College London London UK

**Keywords:** artificial intelligence, automation, business process management, evidence synthesis, machine learning, research waste, systematic reviewing

## Abstract

**Introduction:**

Evidence syntheses are crucial in healthcare and elsewhere but are resource‐intensive, often taking years to produce. Artificial intelligence and machine learning (AI/ML) tools may improve production efficiency in certain review phases, but little is known about their impact on entire reviews.

**Methods:**

We performed prespecified analyses of a convenience sample of eligible healthcare‐ or welfare‐related reviews commissioned at the Norwegian Institute of Public Health between August 1 2020 (first commission to use AI/ML) and January 31 2023 (administrative cut‐off). The main exposures were AI/ML use following an internal support team's recommendation versus no use. Ranking (e.g., priority screening), classification (e.g., study design), clustering (e.g., documents), and bibliometric analysis (e.g., OpenAlex) tools were included, but we did not include or exclude specific tools. Generative AI tools were not widely available during the study period. The outcomes were resources (person‐hours) and time from commission to completion (approval for delivery, including peer review; weeks). Analyses accounted for nonrandomized assignment and censored outcomes (reviews ongoing at cut‐off). Researchers classifying exposures were blinded to outcomes. The statistician was blinded to exposure.

**Results:**

Among 39 reviews, 7 (18%) were health technology assessments versus systematic reviews, 19 (49%) focused on healthcare versus welfare, 18 (46%) planned meta‐analysis, and 3 (8%) were ongoing at cut‐off. AI/ML tools were used in 27 (69%) reviews. Reviews that used AI/ML as recommended used more resources (mean 667 vs. 291 person‐hours) but were completed slightly faster (27.6 vs. 28.2 weeks). These differences were not statistically significant (relative resource use 3.71; 95% CI: 0.36–37.95; *p* = 0.269; relative time‐to‐completion: 0.92; 95% CI: 0.53–1.58; *p* = 0.753).

**Conclusions:**

Associations between AI/ML use and the outcomes remains uncertain. Multicenter studies or meta‐analyses may be needed to determine if these tools meaningfully reduce resource use and time to produce evidence syntheses.

## 1 Introduction

Evidence syntheses such as systematic reviews and health technology assessments (hereafter ‘reviews’) are highly impactful in healthcare, welfare, and other areas, but production is resource‐intensive and can take years. Although the time from initiating to completing health reviews varies greatly [1], fifteen months is typical [2, 3]. Cochrane suggests reviewers should be prepared to spend one to 2 years on a review, but only half are completed within 2 years of protocol publication and median time‐to‐publication has increased [4]. Many reviews—notably those published by Cochrane and health technology assessments in rapidly‐advancing fields such as cancer treatment—need to be updated to include new evidence [5], so resource use often extends beyond first publication. About 25 percent of reviews are outdated within 2 years of publication [6].

### 1.1 Artificial Intelligence and Machine Learning Tools for Evidence Synthesis

Artificial intelligence and machine learning (AI/ML) tools can reduce the need for humans to perform repetitive and complex tasks. “Repetitive and complex” describes several phases of evidence synthesis, such as title and abstract screening against inclusion criteria, extracting data, and assessing risk of bias. Some tasks, such as screening a trial, must be performed potentially thousands of times for each review, often by highly educated experienced researchers. AI/ML tools have been used to automatically screen [7, 8, 9] and classify articles [10], help produce living reviews [11, 12], and systematically map global research on climate and health [13]. AI/ML offers the potential to reduce resource use, produce reviews in less time, help new and inexperienced reviewers learn [14], and maintain or perhaps exceed current expectations of transparency, reproducibility, and methodological rigor.

AI/ML tools have been available to systematic reviewers for at least fifteen years [15]. There is increasing evidence suggesting substantial resources could be saved on specific review phases if AI/ML tools were adopted to aid production [16]. A 2015 review found time savings of 40%–70% in the screening phase when using text mining software [17]; we reported similar or perhaps more (60%–90%) time savings in 2021 [18]. Automatic classification and exclusion of non‐randomized designs with a study design classifier saved Cochrane Crowd from manually screening more than 40% of identified references in 2018 [19]. We have also reported that categorizing studies using automated clustering used 33% of the time compared to manual categorization [18].

Despite this, the evidence synthesis field has been slow to adopt AI/ML [20], although the COVID‐19 pandemic appears to have increased the use of AI/ML tools in evidence synthesis [21]. One explanation for underutilization may be that the field has grown to equate human effort with methodological quality, such that automation may be seen as sacrificing quality [22]. Another explanation may be that too little is known about whether phase‐specific savings translate to resource and time savings for entire reviews.

### 1.2 Evaluating Resource Use and Time‐to‐Completion

The present study had three aims: to assess whether AI/ML use reduces resource use and time from review commission to completion; to provide information for making higher‐level organizational decisions about AI/ML; and help power any subsequent multicenter studies, which we anticipated would be necessary. For the latter reason, we designated the study as a pilot.

Studying the resource used to produce an entire review is important because production is expensive. Studying time‐to‐completion is important because answers to research questions that are late are not useful. It is therefore probably more important to study these two outcomes than savings in individual review phases, which may not ultimately be important. Knowing if and to what degree AI/ML tools reduce resource use and time‐to‐completion could help review producers decide whether to adopt such tools, budget and price their products and services, and understand how project timelines may be affected. A priori, based on the available research that has tended to focus on specific review phases and our own experience, as well as the explicit aims of AI/ML tools, we would hypothesize (i.e., expect) that such tools would reduce resource use and time‐to‐completion.

We are aware of one study that assessed time‐to‐completion (days from pre‐registration to preprint or journal submission) as well as several other outcomes including proxies for resource use [23]. The authors found associations between AI/ML use and publication in higher impact factor journals, more abstracts screened per author and included study, and fewer full‐texts inspected per author, but no difference for time‐to‐completion. However, this study did not attempt to account for endogenous assignment (i.e., reviews were not randomized to use or not use AI/ML), so the findings may be subject to confounding.

## 2 Methods

### 2.1 Setting

We conducted a comparative effectiveness research study using retrospective data as prespecified in a published and peer‐reviewed protocol [24]. The cluster for Reviews and Health Technology Assessments at the Norwegian Institute of Public Health (NIPH) started to adopt AI/ML in 2020 to help map and process large volumes of COVID‐19 evidence. AI/ML use has increased from no use before the pandemic, to 26 reviews after the first year, and nearly all now [18]. A dedicated support team was funded from late 2020 and was tasked with the continuous identification, evaluation (as in this article), and implementation of AI/ML tools to aid review production, and with tailoring them to institutional procedures and processes [18, 25]. Current NIPH standards require AI/ML use, except in rare circumstances that justify a pragmatic deviation. Because most reviews are externally commissioned, detailed resource use information is available, which we analyzed in this study to estimate associations between use of AI/ML and resource use and time‐to‐completion.

### 2.2 Data Collection and Extraction

We obtained a convenience sample of all analyzable reviews on healthcare or welfare topics commissioned at the institute between 1 August 2020 (the first commission to use AI/ML) and 31 January 2023 (administrative cut‐off). A review would not be analyzable if we lacked information necessary for defining any of the necessary variables. RCB sent a list of all potentially eligible projects to the rest of the team and, separately, extracted outcome data, ensuring that the rest of the team were blinded to the outcomes. Systematic reviewer JFME extracted data on the following variables to support the statistical analyses:

- Synthesis type planned (none, such as in scoping reviews; pairwise meta‐analysis or qualitative synthesis; or network meta‐analysis).
- Review type (health technology assessment [HTA] or non‐HTA).
- AI/ML tool used and review phase.
- Field (healthcare or social welfare).

### 2.3 Exposures

The main exposures are AI/ML tool use as recommended by the AI/ML team versus no use. Additionally, we compared non‐recommended use versus recommended use, and no use versus any use. We distinguish between recommended and non‐recommended use because, fifteen months after the AI/ML team was formed, we noticed reviewers using AI/ML alongside, rather than instead of, manual processes. For instance, some reviewers would use a ranking algorithm to screen titles and abstracts, reach the “plateau” indicating all relevant studies have been identified, but then continue to manually screen thousands of remaining and likely irrelevant studies. This would not be expected to offer resource or time savings.

Recommended AI/ML use was defined as AI/ML use in any review phase consistent with the team's guidance (also see Protocol Deviations). Non‐recommended ML was defined as AI/ML use deviating from guidance. The overarching principle we applied to classify exposures was that recommended AI/ML use supplants rather than supplements existing human activity. Non‐recommended AI/ML use was defined as use of AI/ML in any review phase not following guidance (e.g., alongside manual processes). Any AI/ML use was defined as use of AI/ML in any review phase following or not following guidance. No AI/ML use was defined as no use of AI/ML in any review phase.

These definitions result in each review being classified to one or more exposures. For example, a review classified as having used recommended AI/ML would also have used any AI/ML. The number of reviews included in the above comparisons therefore varies.

Classification of exposures was performed by two researchers (JFME and AEM) while blinded to outcome. Classification was performed using final review texts and information recorded in project blogs the AI/ML team used to document support provided to review teams.

The AI/ML tools utilized by NIPH encompassed four broad categories: ranking tools (e.g., priority screening in EPPI‐Reviewer), classification tools (e.g., study design classifier for RCTs and for systematic reviews), clustering tools (e.g., Lingo3G), and AI/ML‐powered bibliographic tools (specifically, OpenAlex).

### 2.4 Outcomes

Resource use was defined as the number of person‐hours recorded against a review from commission until completion or the cut‐off date. The time required by Norwegian commissioners to deliberate on a completed review before allowing NIPH to publish varies between two and 8 weeks and there may be delays that are not recorded by NIPH. We therefore defined a time‐to‐*completion*, rather than time‐to‐*publication*, outcome to prevent introducing unnecessary variance in this outcome. Time‐to‐completion was calculated as the number of weeks from commission to approval for delivery to the commissioner, including time used on peer review; or, for projects ongoing at cut‐off, the number of weeks to the cut‐off date. Ongoing projects were therefore right censored with respect to resource use and time‐to‐completion.

### 2.5 Statistical Analysis

Except as noted in Protocol Deviations, statistical analyses were performed as prespecified in our protocol [24]. The statistician (CJR) was blinded to exposure classification until the analyses were finalized. Because reviews were not randomly assigned to use recommended versus no AI/ML, we planned to model and hence account for endogenous (nonrandom) assignment using the variables field (healthcare or welfare) and prespecification (existence of a protocol). We anticipated that reviews that do not plan to perform meta‐analyses (e.g., qualitative syntheses) use less resource and can be completed in less time. We therefore planned to adjust for planned use of meta‐analysis in all analyses.

Ongoing reviews were right censored at study cut‐off and all analyses accounted for this censoring. We had no reason to suspect informative (nonrandom) censoring, so did not model a censoring mechanism. Resource use was analyzed using extended interval regression [26, 27]. Time‐to‐completion was analyzed using a likelihood‐adjusted‐censoring inverse‐probability‐weighted regression adjustment model [28]. Normality of residuals were assessed using the Shapiro‐Wilk test.

To aid generalization to other institutions, we re‐expressed estimates as relative resource use and relative time‐to‐completion by exponentiating differences in mean log resource use, and by computing ratios of mean times‐to‐completion using the delta method [29]. We present two‐sided 95% confidence intervals and *p*‐values where appropriate and used a prespecified *p* < 0.05 significance criterion throughout. We also summarize the time‐to‐completion data using Kaplan‐Meier estimates of survivor functions (note that these do not account for nonrandom endogenous treatment assignment and are not adjusted). Statistical analyses were performed using Stata 18 (StataCorp LLC, College Station, Texas, USA).

### 2.6 Prospective Risk of Bias Assessment

We used the Risk Of Bias In Non‐randomized Studies of Interventions (ROBINS‐I) tool [30] when writing the protocol to anticipate and mitigate risks of bias, and judged that the study would be at low risk of bias. While ROBINS‐I was developed to be used to assess reported studies, we find this and related tools invaluable for identifying possible methodological limitations at the protocol stage.

### 2.7 Protocol Deviations

It was not possible to model endogenous treatment assignment using both prespecified variables (field and prespecification) in the analyses of resource use because the models did not converge. We therefore used one of the two variables, choosing the variable with the smallest standard error in the assignment model (while blinded). Endogenous assignment of any or recommended AI/ML was modelled by field (welfare reviews were generally more likely to use AI/ML) and recommended AI/ML use was modelled by prespecification (reviews with protocols were generally less likely to use recommended AI/ML).

During data extraction we noticed that there may be two forms of non‐recommended AI/ML use: under‐ and over‐use of AI/ML. We therefore published an updated protocol as a preprint during data extraction but before starting the analysis or unblinding the statistician to redefine the exposures to consider these two forms of non‐recommended AI/ML use [31]. However, too few reviews were judged to have under‐ or overused AI/ML, so it was not possible to run these analyses. We therefore performed and report the analyses as originally planned.

### 2.8 Reporting

We followed the Strengthening the Reporting of Observational Studies in Epidemiology (STROBE) reporting guideline [32] (see Checklist S1).

## 3 Results

### 3.1 Included Reviews

Table 1 summarizes the characteristics of the included reviews. At the protocol stage, we anticipated including around 100 reviews by study cut‐off but were only able to include 39. This was because we were commissioned to produce more ineligible reports than anticipated (e.g., reports that were not reviews, or that planned to use network meta‐analysis) and fewer reviews overall due to budget cuts and downsizing in the aftermath of the COVID‐19 pandemic. Of the 39 reviews, 7 (19%) were health technology assessments versus systematic reviews, 19 (49%) were commissioned on healthcare verses welfare topics, 18 (46%) planned to meta‐analyze, 27 (69%) used any form of AI/ML, and 3 (8%) were ongoing (censored) at cut‐off.

**Table 1:** Characteristics of the included reviews.

	Main exposures		Additional exposures			
	No AI/ML Use	Recommended AI/ML Use	Non‐recommended AI/ML Use	Recommended AI/ML Use	No AI/ML Use	Any AI/ML Use
**Commissioned reviews**	12/39 (31%)	21/39 (54%)	6/39 (15%)	21/39 (54%)	12/39 (31%)	27/39 (69%)
**Completed reviews**	12/39 (31%)	19/39 (49%)	5/39 (13%)	19/39 (49%)	12/39 (31%)	24/39 (62%)
**Review type**						
Health technology assessment (HTA)	3/39 (8%)	2/39 (5%)	2/39 (5%)	2/39 (5%)	3/39 (8%)	4/39 (10%)
Non‐HTA	9/39 (23%)	19/39 (49%)	4/39 (10%)	19/39 (49%)	9/39 (23%)	23/39 (59%)
**Synthesis type planned**						
Any (quantitative or qualitative)	11/39 (28%)	19/39 (49%)	6/39 (15%)	19/39 (49%)	11/39 (28%)	25/39 (64%)
Pairwise meta‐analysis	4/39 (10%)	10/39 (26%)	4/39 (10%)	10/39 (26%)	4/39 (10%)	14/39 (36%)
Network meta‐analysis	0	0	0	0	0	0
**AI/ML used during study identification**						
Ranking	0	19/39 (49%)	6/39 (15%)	19/39 (49%)	0	25/39 (64%)
Classifiers	0	9/39 (23%)	3/39 (8%)	9/39 (23%)	0	12/39 (31%)
Clustering	0	6/39 (15%)	2/39 (5%)	6/39 (15%)	0	8/39 (21%)
OpenAlex	0	5/39 (13%)	0	5/39 (13%)	0	5/39 (13%)
**AI/ML used during data extraction**						
Classifiers	0	0	0	0	0	0
Clustering	0	1/39 (3%)	0	1/39 (3%)	0	1/39 (3%)
Automated data extraction	0	0	0	0	0	0
**Other AI/ML functions**	0	0	0	0	0	0

*Note:* Data are numbers of reviews and percentages of all included reviews.

### 3.2 Association of AI/ML Use With Resource Use and Time‐to‐Completion

**Figure 1:**
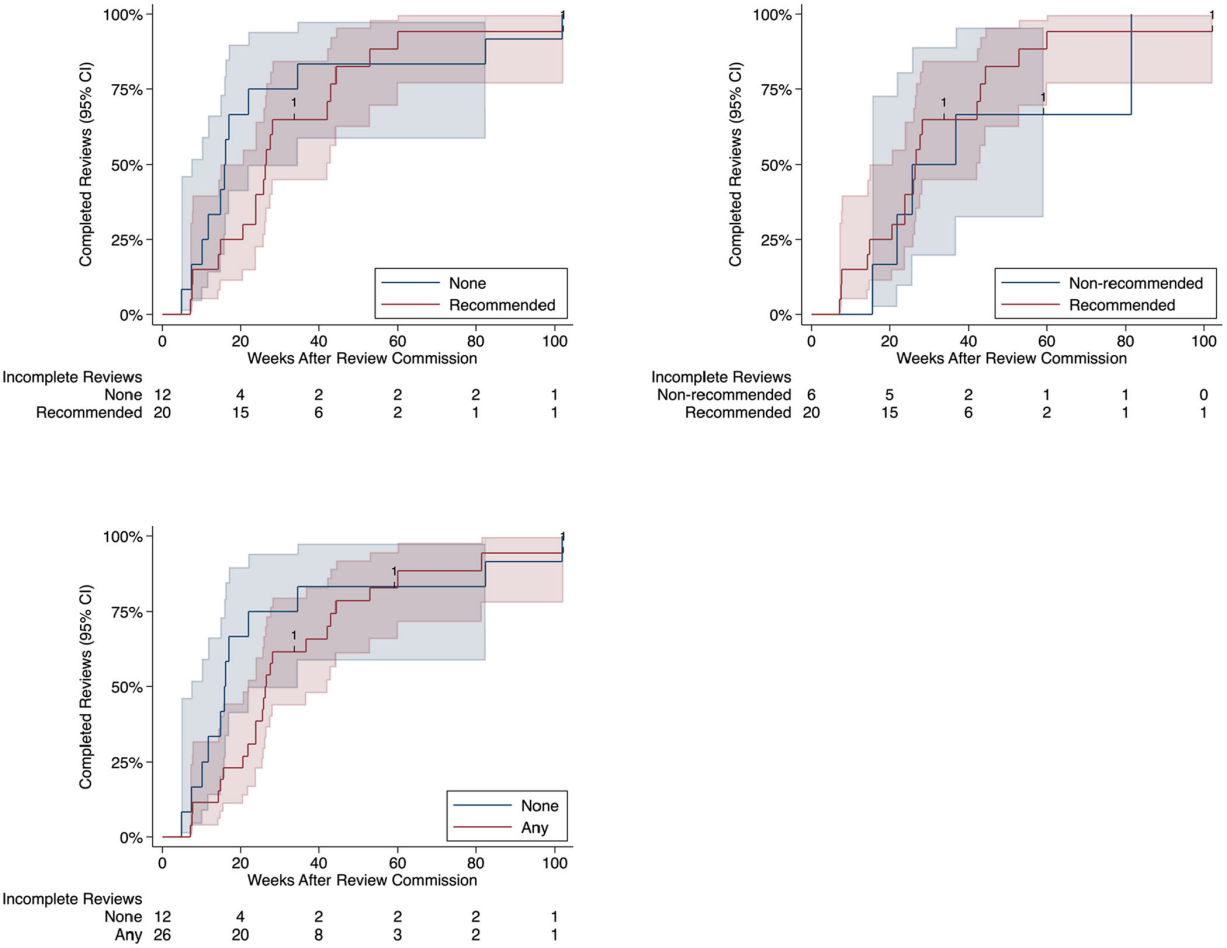
Kaplan–Meier plots for time‐to‐completion. The plots show time‐to‐completion for none versus recommended ML (top left), non‐recommended versus recommended ML (top right), and none versus any ML (bottom), with 95% confidence bands. The estimated survivor functions are unadjusted and do not account for nonrandom endogenous treatment allocation.

The study results are summarized in Table 2. Figure 1 presents Kaplan‐Meier plots for time‐to‐completion. On average, reviews that used AI/ML as recommended used more resources than those that did not (667 vs. 291 person‐hours; relative resource use 3.71; 95% CI: 0.36 to 37.95; *p* = 0.269) but were completed faster (27.6 vs*.* 28.2 weeks; relative time‐to‐completion 0.92; 95% CI: 0.53 to 1.58; *p* = 0.753). None of the effect estimates are sufficiently precise to conclude that use of recommended or any AI/ML is associated with more or less resource use, or longer or shorter time‐to‐completion, compared to no or non‐recommended AI/ML use. For resource use, point estimates favor recommended AI/ML use over non‐recommended AI/ML use, and any AI/ML use over no AI/ML use, while no AI/ML use is favored over recommended AI/ML use. For time‐to‐completion, point estimates favor recommended and any AI/ML use over no AI/ML use, while non‐recommended AI/ML use is favored over recommended AI/ML use.

**Table 2:** Estimates of relative resource use and relative time‐to‐completion.

Exposure (Type of AI/ML Use)	Reviews^a^	Mean (SD)^b^	Effect estimate^c^	*p* value
**Resource use**		Person‐hours	Relative resource use	
**Main exposures**				
No AI/ML Use	12/33 (36%)	291 (379)	3.71 (0.36 to 37.95)	0.269
Recommended AI/ML Use	21/33 (64%)	667 (367)		
** Additional exposures**				
Non‐recommended AI/ML Use	6/27 (22%)	1158 (893)	0.50 (0.02 to 10.74)	0.658
Recommended AI/ML Use	21/27 (78%)	667 (367)		
No AI/ML Use	12/39 (31%)	291 (379)	0.65 (0.22 to 1.93)	0.439
Any AI/ML Use	27/39 (69%)	769 (534)		
**Time‐to‐completion**		Weeks	Relative time‐to‐completion	
** Main exposures**				
No AI/ML Use	12/33 (36%)	28.2 (31.1)	0.92 (0.53 to 1.58)	0.753
Recommended AI/ML Use	21/33 (64%)	27.6 (15.4)		
** Additional exposures**				
Non‐recommended AI/ML Use	6/27 (22%)	36.2 (26.4)	1.12 (0.67 to 1.89)	0.658
Recommended AI/ML Use	21/27 (78%)	27.6 (15.4)		
No AI/ML Use	12/39 (31%)	28.2 (31.1)	0.93 (0.58 to 1.51)	0.784
Any AI/ML Use	27/39 (69%)	29.5 (18.0)		

^a^ Denominators vary by comparison because reviews may not meet the definition of either exposure.

^b^ Data are means (standard deviations) of samples restricted to completed (uncensored) reviews and do not account for nonrandom endogenous treatment allocation.

^c^ Effect estimates account for right‐censored outcomes, nonrandom endogenous treatment allocation, and are adjusted for planned meta‐analysis. An effect estimate < 1 indicates that recommended or any AI/ML use is associated with less resource use or shorter time‐to‐completion than to the comparator.

The estimates of association are generally consistent with the sample means. However, the sample means may be misleading due to possible confounding due to nonrandom exposure assignment, do not account for censoring of ongoing reviews, and are not adjusted for the effect of planned meta‐analysis, which is associated with more resource use and longer time‐to‐completion.

## 4 Discussion

### 4.1 Findings

This study did not identify statistically significant differences in resource use or time‐to‐completion with respect to comparisons between the main or additional exposures. Point estimates for resource use favor recommended over non‐recommended AI/ML, and any AI/ML over no AI/ML use. However, the point estimate for recommended AI/ML versus no AI/ML use favors the latter. Assuming this is correct, we speculate that reviews that did not use AI/ML may have been simpler (could be completed using less resource), more urgent (pressure to use less resource), were perhaps performed less rigorously (therefore using less resource) than those that followed the AI/ML team's recommendations, and that we were unable to account for this in analysis. For time‐to‐completion, point estimates favor recommended AI/ML over no AI/ML use, and any AI/ML use over no AI/ML use. However, the point estimate for recommended versus non‐recommended AI/ML use favors non‐recommended AI/ML use. We have observed two forms of non‐recommended AI/ML use (see Protocol Deviations), one of which would be expected to lead to shorter times to completion and could explain this finding.

It is possible that substantial variance in the overall outcomes we studied is introduced by review phases that were not amenable to automation when the reviews were conducted, such as analysis, GRADEing [33], report‐writing, and peer review. The advent of large language models [34] may represent an opportunity to automate and expedite these phases and hence reduce overall resource use and time‐to‐completion. Other factors, such as differing levels of computer literacy and comfort using AI/ML tools would also be expected to contribute variance that may be difficult to adjust for but are inherent to a task like systematic reviewing that is performed by researchers with diverse educational backgrounds and experience.

### 4.2 Strengths and Limitations

The main strengths of this study are as follows. The study was prespecified and performed according to a published peer‐reviewed protocol, which included a prospective ROBINS‐I risk of bias assessment. The work was conducted with only minor protocol deviations, which we report and justify. We used outcomes that reflect overall production costs and times, which are more relevant than those that focus on individual review phases and which may not translate to overall savings. These outcomes were defined using internal data that are usually not made available. We made three relevant comparisons, emphasizing the use of AI/ML tools according to recommendations. Exposure classification and statistical analyses were performed blind; that said, it is possible the exposure classification blinding was imperfect because JFME was familiar with some of the reviews, so this may have introduced bias. Finally, we used appropriate statistical methods that account for endogenous (nonrandom) exposure assignment and censoring of ongoing reviews.

The main limitations of this study are the retrospective non‐randomized design and the smaller than anticipated sample size. While we prospectively assessed the study to be at low risk of bias [24], we did anticipate that there might be residual confounding that we would not be able to account for in analysis, and this may have occurred. It is possible that some review teams used AI/ML but did not report it in full according to our reporting guidelines, and this might have led to some misclassifications. There were no major protocol deviations, though it was necessary to change how endogenous assignment was modelled for resource use due to non‐convergence, but this analysis choice was made before the statistician was unblinded. We attempted to account for the fact that reviews may have under‐ or over‐used AI/ML, but this was not possible (see Protocol Deviations). We used date of commission to calculate time‐to‐completion, but this may have introduced variance because it is possible that work on some reviews did not start until substantially later. We suggest that future work consider alternative definitions (e.g., date of first literature search). Finally, the administrative cut‐off precluded the inclusion of reviews that had used generative AI tools such as large language models, which were not widely available during the study period, but which we anticipate will have substantial utility in evidence synthesis [35, 36].

### 4.3 Conclusions

The associations between use of AI/ML tools for evidence synthesis and resource use and time‐to‐completion are unclear. Based on the results of this study, we suggest future studies be powered to detect a 30% or better reduction in resource use and a 10% or better reduction in time‐to‐completion. Informal power calculations indicate nonrandomized studies may require a few hundred reviews; randomized trials would be preferable but perhaps infeasible. This suggests multicenter studies or meta‐analyses using nonrandomized evidence will probably be necessary. We suggest that future work study the effect or association of AI/ML on the quality, correctness [36], validity, and reproducibility of reviews, to ensure that the adoption of automation does not lead to suboptimal decision‐making or other harms, and that reviews, review methodology, and stakeholders benefit from new technologies. This study will likely require nonrandomized studies that must carefully address issues such as endogenous (nonrandom) assignment of AI/ML use or nonuse, as in the present paper.

## Author Contributions

**Christopher James Rose:** conceptualization, data curation, formal analysis, investigation, methodology, project administration, supervision, writing – original draft, writing – review and editing. **Jose Francisco Meneses‐Echavez:** data curation, investigation, methodology, project administration, writing – review and editing. **Ashley Elizabeth Muller:** conceptualization, methodology, project administration, supervision, writing – review and editing. **Rigmor C. Berg:** data curation, project administration, writing – review and editing. **Tiril C. Borge:** investigation, project administration, supervision, writing – review and editing. **Patricia Sofia Jacobsen Jardim:** investigation, writing – review and editing. **Chris Cooper:** investigation, writing – review and editing.

## Ethics Statement

The authors have nothing to report.

## Consent

The authors have nothing to report.

## Conflicts of Interest

The authors declare no conflicts of interest.

## Peer Review

The peer review history for this article is available at https://www.webofscience.com/api/gateway/wos/peer-review/10.1002/cesm.70030.

## Reporting Guideline

We followed the Strengthening the Reporting of Observational Studies in Epidemiology (STROBE) reporting guideline (see Checklist S1).

## Protocol

Muller AE, Berg RC, Meneses‐Echavez JF, et al. The effect of machine learning tools for evidence synthesis on resource use and time‐to‐completion: protocol for a retrospective pilot study. *Syst Rev*. 2023;12(1):7, https://doi.org/10.1186/s13643-023-02171-y.

## Supporting information

STROBE‐checklist‐v4.docx.

## Data Availability

Data and analysis code are available at https://doi.org/10.5281/zenodo.14616537.
